# Metabolites of Life: Phosphate

**DOI:** 10.3390/metabo13070860

**Published:** 2023-07-19

**Authors:** Janusz Wiesław Błaszczyk

**Affiliations:** Jerzy Kukuczka Academy of Physical Education, 40-065 Katowice, Poland; j.blaszczyk@awf.katowice.pl

**Keywords:** life cycle, energy metabolism, phosphate, bone, aging

## Abstract

The process of aging and escalating the failure of all body organs has become the center of interest in contemporary science and medicine. The leading role of phosphate-calcium tandem deficiency as a pacemaker of metabolic senescence has emerged recently. Most of the phosphates in the human body are stored in the bones, which seem to play a pivotal role in all metabolic and energetic processes. Bone metabolism combines physical activity with adaptive changes in the internal environment of the body, which is necessary for its survival. Phosphate-calcium signaling is the primary mechanism for controlling homeostasis and its recovery after exercise-induced disorders. Phosphates play an important role in the regulation of energy metabolism both by regulating postprandial glucose storage in the muscles and in the liver, as well as the distribution and adaptation of energy metabolites to the needs of the brain and skeletal muscles. The bone-driven energy metabolism is of decisive importance for maintaining all vital functions of the body organs, including their proper functioning and integrated interplay. The phosphate-calcium tandem contributes to the development and proper functioning of the organism, whereas energy dysmetabolism is the main cause of aging and the final termination of life.

## 1. Algorithm of Life

The process of systemic aging and the related deficiency of all body organs has become the center of interest in modern science and medicine. The leading role of energy dysmetabolism in involutional processes has been increasingly recognized [1]. In this regard, the role of glucose metabolism dysfunction has been quite well explained [1]. However, many details concerning the mechanisms that drive aging-related changes in energy metabolism have not yet been explained. Recently, a groundbreaking hypothesis emerged postulating the leading role of phosphate-calcium tandem deficiency as a pacemaker of metabolic senescence [2,3].

Systemic energy metabolism relies firstly on two main substrates: glucose and oxygen [1]. Cellular life is maintained by oxidative phosphorylation, which uses, on average, six molecules of oxygen per every molecule of glucose [4]. To satisfy these needs, under physiologic conditions, the oxygen and glucose extraction from blood should also be balanced [4]. Taken together, the vital function of the circulatory system is a continuous supply of balanced amounts of energy metabolites to all body cells to maintain homeostasis. Moreover, systemic energy control must also quickly respond to dynamic changes in energy consumption in skeletal muscles during physical activity. Therefore, the second vital role of energy metabolism control is to protect the brain from energy-fuel deficiency. The increased levels of energy consumption by the skeletal muscles cannot compromise brain functioning. The aim of maintaining a stable supply of energy fuels to the brain and the entire nervous system, as well as the heart and respiratory system, has emerged as avital task for energy metabolism control. Without any doubt, oxygen and glucose delivery to the brain, heart, and the muscles of the diaphragm is a priority for the control of energy production and distribution. The energy distribution to strategic organs is particularly challenging in the case of intensive physical activity when most of the energy fuel must be allocated to the activity of striatal muscles. To solve this energetic dilemma, the intake of glucose by the striatal muscle is mediated by ATP produced by the heart, muscle, and bone tissue. Systematic energy control monitored energy fuel distribution online and reorganized it according to the needs of the muscular system. The fundamental role in these processes seems to be played by the phosphate-calcium tandem [2,3].

**Figure 1:**
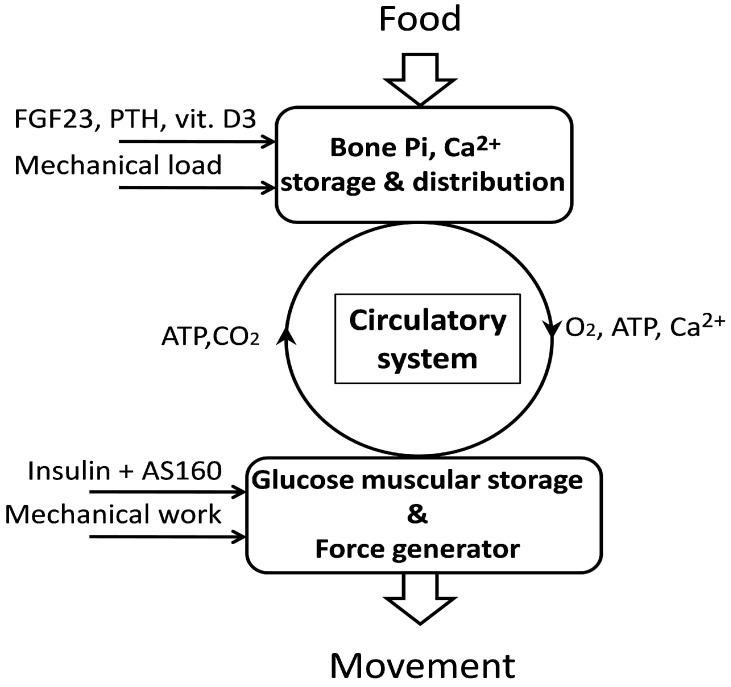
Muscle–bone interaction in the processes of energy metabolism, in which the body’s phosphate-calcium homeostasis plays a fundamental role. Calcium and phosphorus obtained from the food you eat are stored in bone tissue, while glucose is stored in skeletal muscles, creating autonomous energy resources in the muscles. Motor activity is a factor that integrates both processes and regulates their level. A detailed description is given in the text.

**Figure 2:**
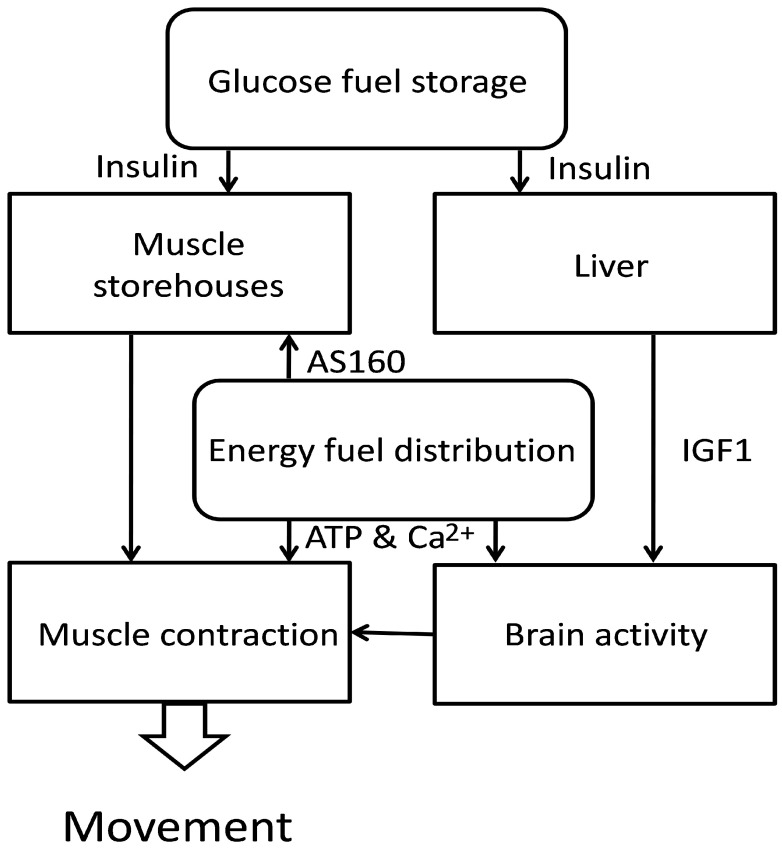
Distribution of glucose energy resources between the nervous system and skeletal muscles. Due to the high energy consumption inskeletal muscles during movement, they are supplied by the glycogen accumulated after meals. The brain, on the other hand, uses glycogen stored in the liver, which remains under control by pancreatic hormones. Additionally, the liver, in response to parathormone, releases IGF1, which allows the brain to increase its use of glucose fuel. For details, see the text.

The total energy necessary to maintain the vital functions of the whole organism, especially in conditions of increased activity, can exceed the level of energy reserves obtained from foods, and a chronic energy crisis could pose a threat to health and life. Therefore, the human body has been equipped with a mechanism that controls the entire energy metabolism and is supported by the mechanism of selective energy distribution shown schematically in Figure 1 and Figure 2. Both mechanisms are based on phosphate signaling. Thanks to these, the supplies of energy fuel, especially glucose, and oxygen, can be distributed between individual tissues and organs according to the level of their metabolism and current activity. Such control requires the fast and effective relocation of energy resources to organs and tissues that are distinguished by high dynamics of activity which are usually necessary to sustain the life of the organism. In particular, the brain and muscles are provided with physiological mechanisms that allow for a rapid increase in energy supply, especially during the transition from the resting to the active phase. This transition to the active phase in the brain, and can be associated with the need for a temporary reorganization of energy distribution in the body. Limited energy resources imply that increased portions of energy are directed first to the stimulated areas of the brain. 

The skeletal muscles are the major energy consumers, and thus, selective energy supply could be limited to the muscle groups that realize the current motor behavior. Moreover, the post-exercise recovery of muscle and brain tissue recovery of homeostasis can still also be associated with additional energy expenditure [1]. The output measure of cellular respiration is the level of ATP produced by the tissue. In both organs, a deficient energy supply strikes the ATP production immediately, and the resultant drop in phosphates reorganizes the systemic energy metabolism that aims to recover energy balance. In the case of an acute energy crisis, lowered levels of blood phosphate inhibit the activity of the striatal muscles, which are the main energy consumers. This phosphate-dependent safety procedure also utilizes calcium ions as an energy metabolism cofactor. Consequently, the systemic use of oxygen, glucose, phosphate, and calcium are mutually dependent, and their levels in physiological conditions can be very precisely controlled.

To maintain energy balance and sustain life, the operation of the energy quadriga consisting of oxygen, glucose, calcium, and phosphate, must be precisely coordinated. In this tandem, the primary controlling mechanism of glucose distribution and storage is based on the Akt substrate regulating glucose transporter (GLUT4) translocation (AS160). This substrate links insulin and insulin-like growth factor (IGF1) signaling and glucose uptake and storage in the muscles, liver, and adipose tissue [1,5]. The postprandial rise in blood glucose triggers the release of insulin, which controls the systemic targeted distribution and storage of glucose [1,5]. With active muscles being the main consumer of energy, they are also the largest autonomic stores of glucose [6,7]. In the process of energy metabolism, the AS160 phosphorylation operates as a metabolic valve that activates the glucose transporter GLUT4 both in skeletal muscle and adipocytes. The postprandial release of insulin results in almost a three-fold increase in AS160 phosphorylation. Additionally, physical activity intensifies AS160 phosphorylation in all active muscles and the heart [5]. Most importantly, efficient active muscles, and especially the heart, are the main source of ATP circulating in the blood, which synchronizes the activity of all tissues, including the brain. The age-related dysfunction of the AS160 phosphorylation valve limits muscle access to glucose and impairs its storage in the process of glycogenesis [8]. These seem to be the main pathomechanism of peripheral insulin resistance that disorganize muscle energy metabolism and physical activity. As a result of a growing energy deficit, motor activity in older adults declines gradually, leading to the development of osteoporosis and sarcopenia, both escalating muscle metabolic dysfunctions culminating in rhabdomyolysis [5], which seems the final rescue trial when balancing life-sustaining energy metabolism.

We are only at the beginning of the way of learning about multi-level and multi-threaded phosphate control mechanisms. In this narrative review, I focus on phosphate physiology, its changes in aging, and some other pathologies.

## 2. Physiological Roles of Phosphate

Phosphates play a leading role in energy metabolism. The energy necessary for the functioning of the brain and muscular system can be obtained at the cellular level in mitochondrial oxidative phosphorylation processes. During this complex process, glucose oxidation provides the energy that is necessary to obtain adenosine triphosphate (ATP), which is an intracellular source of ionized phosphorus, and the energy necessary for the implementation of numerous life processes [1]. Depending on energy requirements, the cells of the human body can be divided into two populations. The first of them is characterized by a rather stationary energy metabolism, the level of which in an adult organism change slowly and to a small extent in a circadian rhythm. The second exceptional population consists of active cells that are characterized by high dynamics of metabolic and energetic processes. This group includes neurons, but above all, the myocytes that make up muscle tissue. In the first case, the life and function of the neurons, especially of the cerebral cortex and memory networks, require large amounts of energy [9]. However, it is the functioning of the motor system, consisting of nearly 650 skeletal muscles, that is the greatest challenge for the entire energy management of the body and thus has a decisive impact on our lives [10]. At rest, the functioning of such organs as the heart or diaphragm muscles requires a relatively constant but life-long uninterrupted supply of energy. In skeletal muscles, the dynamics of energetic processes are quite different. Their anatomic structures and level of activity are very diverse. While resting energy consumption in the muscular system remains at a similar level as in the brain, the situation changes critically in cases of increased physical activity [11]. When skeletal muscles rapidly increase their energy consumption, they meet the demands required by the restricted energy supply to other tissues, including the brain. Eventually, this can lead older individuals to an energy conflict that may pose a threat to their life [12].

Although adenosine triphosphate (ATP) is commonly known as the energy source for intracellular metabolic functions, extracellular ATP plays a vital role as a signaling molecule in a variety of physiological processes [13]. ATP provides the energy for muscles to generate force. The regulation of calcium (Ca^2+^) during contractions and the phosphorylation of the myosin light chain has a significant impact on ATP usage [11]. The use of ATP through these mechanisms is sufficiently high enough that muscles can quickly deplete ATP [11]. At rest, skeletal muscles have a low metabolic rate, and the intracellular concentration of ATP does not exceed 7 mM [11]. Action potentials on the membrane are translated into a release of Ca^2+^ that initiates a muscle contraction and, as a result, increases ATP demand through the activity of the myosin and Ca^2+^ ATPases [12,14]. Muscle activation can increase the muscle’s ATP requirement by more than 100 times [11]. To protect the muscle from energy deficiency, in active muscles, ATP is massively produced by the degradation of glycogen. In the process of glycogenolysis, glycogen reserves are stored in the muscles, which are catabolized by the sequential removal of glucose monomers via phosphorolysis. This reaction takes place in the myocytes and is under the regulation of two key enzymes: phosphorylase kinase and glycogen phosphorylase. 

The process of glycogenolysis supplies the necessary amounts of pyruvate for oxidative phosphorylation into the mitochondria. In the muscle cells, the Ca^2+^ concentration regulates the rate of ATP use by the Ca^2+^ATPase and myosin ATPase activity, which together account for 90% of ATP consumption [11]. Regulating Ca^2+^’s release from the sarcoplasmatic reticulum, and thus the intracellular Ca^2+^ concentration that dictates the rate of ATP use in the muscle cell, these highly redundant control mechanisms provide an effective means by which ATP can be preserved at the cellular level, avoiding metabolic catastrophe [11]. 

The human diet is a rich source of phosphorus and calcium. A large supply of these metabolites could cause large fluctuations in the level of these metabolites in the blood, which interfere with their functions. Therefore, bone tissue forms a buffer and a boundary store separating the outer and inner areas of phosphate and calcium metabolism. Calcium ions and phosphates gain their physiological properties only after they are released from bone storage.

There are several hundred grams of phosphate in the adult human body. Most of it (85%) is present in the bone or teeth as hydroxyapatite, and about 15% is present within cells. Extracellular inorganic phosphate constitutes barely 1% of the total phosphate [15,16]. In healthy adults, several hundred milligrams of phosphate are daily absorbed in the intestine, and nearly the same amount is excreted into the urine, thereby maintaining a phosphate balance [15,16]. Diet-derived phosphate can be absorbed into the bones, which forms the systemic storage of phosphate. Several biochemical mechanisms are involved in the regulation of bone phosphate storage. The main operation is based on vitamin D, parathyroid hormone (PTH), and fibroblast growth factor 23 (FGF23) [15,17]. Plasma phosphate concentration is age dependent. In children, there is a positive balance of 2–3 mmol/day, and in elderly subjects, there is a small negative balance, primarily due to age-related bone loss [17]. 

To accomplish its physiological functions, serum phosphate needs to be precisely controlled. The serum phosphate level is regulated by intestinal phosphate absorption, renal phosphate handling, and the equilibrium of extracellular phosphate with bone or in intracellular fluid.PTH, vitamin D, and FGF23 regulate serum phosphate by modulating intestinal phosphate absorption, renal phosphate reabsorption, and bone metabolism. Additionally, glucocorticoids are suppressed while the growth hormone (GH) enhances renal phosphate reabsorption by modulating the expression of sodium–phosphate cotransporters. Physiologically, extracellular concentrations of phosphate and these hormones are maintained by several negative feedback loops [15]. Lowering the content of calcium ions in the blood causes the parathyroid glands to release PTH, which affects the bones and kidneys directly. PTH increases renal calcitriol synthesis, which together, increases the reabsorption of calcium into the kidneys and reduces the urinal excretion of calcium. At the same time, calcitriol increases the intestinal absorption of calcium and phosphorus. Calcitriol also has an inhibitory effect on the activity of the parathyroid glands by reducing PTH synthesis. Importantly, PTH can also increase the osteoclast number and thus amplify the osteoclast function to increase bone resorption and the release of calcium from the bone to the blood.

## 3. Phosphate-Calcium Tandem

In the human body, calcium and phosphate metabolism are closely related [2,3]. Phosphates are counterions of calcium, which ensures their compounds and electrical neutrality. Both phosphate and calcium ions play a key role in neuromuscular control. Their concentration determines the activity of muscle and nerve cells, the permeability of cell membranes, and the activity of some enzymes. Importantly, mechanical stress in bones due to physical activity generates mechanosensory signals which stimulate osteoblasts to absorb and osteoclasts to release balanced quantities of phosphate and calcium [18].

The calcium–phosphate tandem coordinates energy metabolism throughout the body. It has a fundamental effect on glucose metabolism, its accumulation in muscle stores, in the liver, and in adipose tissue. Calcium ions are involved in many vital functions in the human body. They are necessary for the stabilization of all processes of biological activity of glandular, nervous, and muscle cells. They are involved in the release of mediators, ensuring the primary synaptic transmission of nerve signals. They also participate in muscle contraction. Calcium ions are responsible for adjusting the metabolism of nerve and muscle cells to the level of their activity. To this end, calcium ions increase the activity of the mitochondria in neurons and glial cells, especially oligodendrocytes and myelin, to increase the metabolism of the tissue. Calcium-phosphate signaling creates a complex system of secondary transmissions on which the energy balance of the body and its tissues depends. Such vital functions require maintaining a constant level (homeostasis) of calcium and phosphate ions in the blood. Thus, it is reasonable to claim that the ATP and Ca^2+^ tandem controls life and death [2,3].

Slowly progressing through a decline in the level of calcium and phosphorus ions may even be asymptomatic for a long time. Low calcium levels (hypocalcemia) are signaled by paraesthesia, itching, pricking, or tingling around the mouth and distal parts of the limbs: the hands and feet. These symptoms are probably related to the increased excitability of the nervous system and peripheral nerves. Another symptom of hypocalcemia is painful spasms (tetany) in the muscles of the arms and legs. In addition, the patient may complain of chronic fatigue, sleep disorders, headaches, bone pain, and abdominal pain. Importantly, hypocalcemia is usually accompanied by an increase in phosphate levels above 1.4 mmol/L, i.e., hyperphosphatemia.

## 4. Blood Transport and Distribution of Phosphate

A special role in the implementation and optimization of energy processes is played by phosphates and their capability to store energy in chemical bonds of adenosine triphosphate. It has been estimated that during the day, the human body processes the amount of ATP comparable to its body weight [19]. This fact, combined with the complex processes of energy metabolism control, suggests that phosphates are the main link in metabolic processes that have a decisive impact on the life and death of an organism [19]. Although our knowledge on this subject is still incomplete, the research results collected so far support this hypothesis. 

The level of ATP circulating in the blood is the result of the collective, closely coordinated activity of all body organs, however, the leading role is played by the activity of the heart. Contractions of the heart muscle, whose mitochondria constitute as much as 40% of its mass, are the main source of plasma ATP. Importantly, about 10% of the blood is directed to the bone marrow, which is the site of the integration of phosphate and calcium signaling [20]. Depending on the level of glucose, oxygen, and carbon dioxide in the blood, the bones adjust the level of activity of osteoclasts and osteoblasts. 

The blood supply to the bone is delivered to the endosteal cavity by nutrient arteries and flowing through marrow sinusoids before exiting via numerous small vessels that ramify through the cortex [20]. The marrow cavity affords a range of vascular niches that are thought to regulate the growth and differentiation of hematopoietic and stromal cells, in part via gradients of oxygen tension. The quality of the vascular supply to the bone tends to decline with age and may be compromised in common pathological settings, including type 2 diabetes, anemias, and immobility [20]. A reduction in the vascular supply is associated with bone loss. Hypoxia inhibits osteoblast function and bone formation while simultaneously causing reciprocal increases in osteoclastogenesis and bone resorption [20]. Common regulatory factors such as the parathyroid hormone (PTH) and NO are potent vasodilators and might exert their osteogenic effects on bone via the vasculature [20]. Additionally, mechanical stress in bones due to physical activity stimulates osteocytes to absorb calcium and inhibits the osteoclasts’ release, resulting in balanced levels of phosphate and calcium.

Respiration can have a significant impact on blood phosphate levels [14]. Respiratory alkalosis due to hyperventilation causes a reduction in phosphate excretion, whereas respiratory acidosis (due to hypoxia or excessive level of blood CO_2_) has the opposite effect. Respiratory alkalosis and increased plasma glucose result in hypophosphatemia (serum phosphate concentration < 2.5 mg/dL) [21]. Both respiratory and metabolic alkalosis causes a decrease in serum phosphate; however, the effect is greater with respiratory alkalosis. Respiratory alkalosis causes intracellular CO_2_ to decrease, resulting in intracellular alkalosis, which increases glycolysis via the stimulation of the key glycolytic enzyme phosphofructokinase. Serum phosphate falls within 20 min of hyperventilation, and it persists for 90 min after the ventilation returns to normal. Even small amounts of intravenous glucose can cause significant hypophosphatemia. If it is accompanied by hyperventilation, the fall in serum phosphate is greater. The resulting hypophosphatemia is common in the elderly, and what is most important is that each additional increase in phosphate loading to the erythrocytes could deepen the metabolic crisis leading to cardiac arrest and respiratory muscle failure in the diaphragm [14,22].

Ten percent of the circulating blood passes through the bone marrow to monitor the efficiency of the blood-based transport of energy metabolites, i.e., oxygen, phosphate, calcium, and glucose. Bone marrow allows for the supply of these metabolites to be adjusted depending on their use and the capability of the blood to transport them. Towards this aim, the bone marrow senses the level of calcium, oxygen, and phosphate, as well as some hormonal signals [23]. It responds to any changes through the regulation of hematopoiesis. Changes in intracellular calcium levels can regulate the proliferation and differentiation of bone marrow stem cells, promoting osteogenesis, angiogenesis, and chondroblast differentiation. Hematopoietic stem cells derive regulatory information from the bone [23]. Parathyroid hormone (PTH) stimulated osteoblastic cells that increase in number produce high levels of the Notch ligand jagged 1 and support an increase in the number of hematopoietic stem cells [23]. Ligand-dependent and PTH coactivation increases the number of osteoblasts. Osteoblastic cells are a regulatory component of the hematopoietic stem cell niche that influences stem cell function [23]. Aging is associated with the lower osteogenic and greater adipogenic biasing of hematopoietic stem cells. Hormonal changes in aged women have been associated with an accelerated age-related increase in sympathetic activity, which has been reported to be a primary cause of osteoporosis. Depression is another neural condition in which an increased sympathetic tone could contribute to osteoporosis [24].

Erythropoietin (EPO) is a glycoprotein cytokine secreted in adults mainly by the kidneys in response to hypoxia. It stimulates erythropoiesis in the bone marrow. Low levels of EPO are continuously secreted to compensate for normal red blood cell turnover. Hypoxia and interstitial fibroblasts in the kidney increase the release of EPO, thus accelerating the production of new cells and boosting the population of erythrocytes. When oxygen levels exceed physiological needs, fibroblasts lower EPO production, and the population of erythrocytes decreases [25]. Hyperventilation can cause the selective removal of even young erythrocytes [25].

The level of oxygen, CO_2_, and ATP also has a significant impact on hematopoietic processes in the bone marrow. The continuous, lifelong process of replacing worn-out erythrocytes with new ones that are rich in ATP can be controlled by the level of ATP and other energy metabolites in the blood. In immature erythrocytes (reticulocytes), the intracellular phosphate level is set by both the transient mitochondrial activity that is triggered and supplemented by the translocation of plasma ATP independently of insulin [26]. Recently, it has been documented that electromagnetic stimulation can regulate the proliferation and differentiation of bone marrow stem cells, promoting osteogenesis, angiogenesis, and chondroblast differentiation [27]. The regulation of channels, transporters, and ion pumps by electromagnetic stimulation induces calcium oscillations, promoting bone and cartilage repair and inhibiting tumor stem cell growth [27]. The opposite impact of the electromagnetic field on normal and tumor stem cells is likely related to the specificity of the tumor stem cells’ calcium channels [27].

Erythrocytes or red blood cells (RBCs) are an important source of ATP in the vasculature of active tissues. A mature red blood cell is biconcave that is disc-shaped with a diameter of 8 microns [28]. They are capable of extreme changes in shape and, due to their flexibility, can easily squeeze through capillaries much narrower than their diameter and can almost immediately recover their original shape. ATP released into arterioles causes vasodilation, thus increasing the volume of blood perfusion.

Phosphates released from bones are transported by the blood to all tissues, where they participate in the multiple processes of protein phosphorylation and energy metabolism and act as an energy booster. Especially in the brain, glucose phosphorylation allows it to be used as an energy source in the process of oxidative phosphorylation. In human red blood cells, the decline in intracellular phosphate attenuates 2,3-bisphosphoglycerate (2,3DPG) activity which adjusts the affinity of hemoglobin to oxygen and its release of near tissues that need it most.

The main factor that determines systemic phosphate metabolism is the condition of the blood tissue. Erythrocytes are very peculiar cells. Their number in the blood is precisely adjusted to the needs of the body, and especially to the need to provide the right amount of oxygen to the brain and muscles [29]. Mature erythrocytes are devoid of cell nuclei and mitochondria; therefore, their lifespan is limited to 120 days [28,29,30,31]. Senile and inefficient erythrocytes are removed from the bloodstream in the process of exocytosis, and new ones appear in their place, resulting from the division of hemopoietic stem cells in the bone marrow. The process of the division and differentiation of erythrocyte precursors lasts about 7 days, of which, within the last 24 h, already after entering the bloodstream, mature erythrocytes lose their internal organelles. Only for one day, the new mature erythrocytes, constituting only 1% of the population of the red blood cells, are equipped with mitochondria and are capable of producing ATP. For this short time, the erythrocytes can deliver the ATP to the muscles and brain. The efficiency of the phosphate transport and distribution by the blood guarantees the massive production of erythrocytes, reaching a level of 2.5 million cells per second [29].

Erythropoiesis is a complex process that occurs in the bone marrow and involves the differentiation of multipotent hematopoietic stem cells to mature enucleated erythrocytes. The human body generates about 2 × 10^11^ new erythrocytes daily through the process of erythropoiesis [32,33]. Approximately 1% of circulating erythrocytes are cleared and replaced by new cells daily [32]. The reticulocytes reach the bloodstream, where they mature by losing their internal organelles, remodeling their plasma membrane, and finally becoming red blood cells. 

The process of erythropoiesis described in simple numbers does not look very impressive; however, if we realize that each new cell becomes a carrier of ATP signals, we can see how important this process is for the health and life of the body. The constant massive exchange of ATP carriers, in combination with a relatively short half-life, allows the maintenance of metabolic processes at a constant level for many years. The exchange of erythrocytes is dictated by their mechanical wear. The procedure of releasing oxygen and ATP molecules carried by erythrocytes relies on pumping erythrocytes with a diameter of 8 μm through arterioles forming the vascular bed of the brain, muscles, and bones, with a diameter that is half that. After each such mechanical test, cells that were unable to recover their native form were removed through phagocytosis in the spleen and liver. During pumping, the surface of the erythrocyte cell membrane slowly wore off, and these irreversible changes in membrane composition triggered the process of phagocytosis.

In the face of the fact that mature erythrocytes are enucleated and without mitochondria, it can be posited that the majority (99%) of circulating RBCs are simply passive transporters of oxygen and CO_2_, 2,3-DPG, ATP, inositol, and other organic phosphates, which bind to hemoglobin and decrease its affinity for oxygen. Particularly, 2,3-DPG levels in the erythrocytes help regulate hemoglobin oxygenation. The unloading of oxygen in tissue capillaries is increased by 2,3-DPG, and small changes in its concentration can have significant effects on oxygen release. 

In humans, 2,3-DPG is the most abundant phosphate compound in the red cell and is the principal allosteric effector for hemoglobin. It is formed by the rearrangement of 1,3-bisphosphoglycerate: an intermediate in glycolysis. 2,3-DPG is hydrolyzed to 3PG by the phosphatase activity of phosphoglycerate mutase (PGM) when stimulated by glycolate-2-phosphate. This reaction can be linked to the conversion of NADH to NAD. Both protons (H^+^) and CO_2_, which are heterotropic effectors of hemoglobin to lower its pH from 7.4 to 7.2, are involved in the increased release of oxygen.

## 5. Phosphate and Muscle Activity

The next control mechanism of the body’s energy metabolism is the distribution of the energy fuel that is optimized and adapted to the level of tissue activity. These mechanisms are controlled by calcium-phosphate signaling. The control of muscle contractions is a critical function in the cardiovascular system, and abnormalities may be life-threatening [12]. In muscle contractions, the interaction between the protein filaments myosin and actin is primarily regulated by intracellular Ca^2+^. The phosphorylation of the myosin regulatory light chain (RLC) is a primary molecular switch for smooth muscle contractions [12]. The equilibrium between phosphorylated and unphosphorylated RLC is dynamically achieved through two enzymes: myosin light chain kinase, a Ca^2+^-dependent enzyme, and myosin phosphatase, which modifies the Ca^2+^ sensitivity of contractions. In cardiac muscle, the primary target protein for Ca^2+^ is troponin C on thin filaments; however, RLC phosphorylation also plays a modulatory role in contractions [12]. Active skeletal muscle can increase its rate of ATP use by more than 100 times [11]. A high rate of ATP use cannot be sustained in the muscle, and ATP use must thus be regulated.

In healthy human beings, blood flows to the brain, and the contracting skeletal muscle is regulated primarily to match the oxygen delivery closely with utilization [10]. The red blood cells (RBCs), the primary O_2_ carriers in the blood, contribute to the regulation of the local processes that match O_2_ supply and demand [10,30]. RBC’s capability to release the ATP increases in response to reductions in erythrocyte and plasma O_2_. The released ATP binds to P2Y purinergic receptors in the vascular endothelium, thereby stimulating the production of endothelial nitric oxide and endothelial-derived hyperpolarization factors, which, in turn, cause local vasodilatation [10].

Glycogenesis is the process of glycogen synthesis, in which glucose molecules are added to chains of glycogen for storage [34]. Glycogenesis contributes to maintaining normal blood glucose but simultaneously creates local reserves of easy-accessible and efficient fuel for muscle contractions since the muscle can utilize the phosphorylated form of glucose directly [34]. Importantly, the capacity and efficiency of muscular glucose storage is critically dependent on muscle activity. Inactive muscles do not use the stored glucose and, thus, cannot accept new portions of sugar retrieved from food. This is the main physiological disturbance leading to insulin resistance and an elevated blood sugar level.

Glycolysis and the local fuel supply to skeletal muscle are initiated by muscle activity. Normally, there are about 400 g of glycogen stored in the muscle creating a muscular protective energy buffer. Consequently, despite the rapid rise in glucose use in the working muscles (up to 10-fold), the muscular energy buffer allows the avoidance of systemic energy crises [6]. This potential crisis would strike first the brain, which is the most energy-sensitive organ and permanently consumes about 60% of blood glucose. Therefore, proper brain functioning is protected additionally by the liver energy buffer, which stores about 100g of glucose [6,7]. The excess energy fuel is stored in adipose tissue.

Intravascular ATP might be an important mediator in local metabolic sensing and signal transduction between the RBCs and the endothelial and smooth muscle cells in the vascular beds of skeletal muscle [10]. The ATP release can also be elevated in conditions of increased body temperature, hypoxia, hypercapnia, and obstructed blood flow (e.g., due to elevated blood flow resistance) [10]. Blood flow in active skeletal muscles increases with the elevations of the work they perform. The magnitude of increase in skeletal muscle perfusion from rest to maximal exercise in normal conditions can reach 100- to 160-fold [10].

## 6. Phosphate Signaling in the Brain

Brain activity also depends on the balance between neuronal activity and its metabolic demands [35]. Given the dynamic nature of brain activity and the considerable metabolic needs of bioelectrically active nervous tissue, the microcirculation of the brain must be highly responsive to the tissue it supplies [35]. This neuronal function also depends on Ca^2+^/GABA mechanisms. The n normal level of GABA helps maintain the tightness and selectivity of the blood–brain barrier [35]. 

Astrocytes represent a logistic arm of the brain, assuming full homeostatic control over the central nervous system’s development and functions [36]. Ca^2+^ signaling is thought to play an important role in synaptic plasticity, memory, cognition, sleep, and behavior [36,37]. The stimulation of astrocytes with ghrelin modifies glutamate and glucose metabolism as well as glycogen storage by decreasing GLUT2, glutamine synthetase, and lactate dehydrogenase, and increasing glutamate uptake, glycogen phosphorylase, and lactate transporters, which might modulate the signals/nutrients reaching neighboring neurons [37].

Insulin-like growth factor 1 (IGF1) controls a variety of cellular processes, such as growth, differentiation, and the maintenance of cell stationary metabolism [38]. Thisis produced mainly in the liver under growth hormone (GH) control. IGF1 mediates anabolic biological processes, including an increase in glucose metabolism, glycogenesis, lipid and protein synthesis, and the inhibition of gluconeogenesis, lipolysis, and protein degradation. All these processes contribute to memory network formation in the brain [9]. After reaching adulthood, the systemic production of GH and IGF1 declines, steadily reaching very low levels in humans aged 60 years [39]. Decreased IGF 1 in middle-aged bone marrow induces myeloid-biased hematopoiesis. It is claimed that, in young individuals, lower systemic GH and IGF1 levels could impact their lifespan by slowing aging processes [39,40]. Interestingly, fasting or dietary restrictions also lower circulating IGF1 levels and have beneficial effects on hemopoietic stem cell function [41,42]. Hemichannels formed by protein subunits called Connexins 43 (Cx43) are major pathways for intercellular communication in the brain [43]. Leaky hemichannels exchange Ca^2+^ continuously, resulting in cell apoptosis. The dysfunction of the astrocyte Cx43 hemichannels results in neuronal network malfunctioning and death. In ischemic episodes, astrocytes open their Cx43 hemichannels, probably due to dephosphorylation [44]. This induces a massive release of ATP and glutamate from astrocytes which opens Pannexin1 channels in neurons, resulting in their death [44].

In humans, the subventricular zone (SVZ) is a specialized area containing neural stem cells that generate new GABA fast-spiking interneurons in the striatum throughout life [35]. The proliferative stem cells of the SVZ migrate and form interneurons in the adjacent striatum, shaping motor memory [35]. The ability of these cells to migrate to the destined tissues is crucial for memory formation. Cytosolic Ca^2+^ is a primary second messenger in the control and regulation of a wide range of cell functions, including cell migration [45]. Contacts between SVZ precursors and blood vessels are unusually permeable and are frequently devoid of astrocyte and pericyte interferences, suggesting that blood-derived cues, including ATP, are gaining direct access to adult neural precursors and their progeny [46,47].

The failure of erythropoiesis in older adults leads to the proliferation of damaged and NAD-deficient red blood cells. These cause the greatest damage and metabolic deficits in tissues with the largest vascular bed, particularly in the brain and skeletal muscles. In the brain, this pathological state of erythrocytes leads to the progressive damage of the blood–brain barrier (BBB) [48]. The alterations and breakdown of functional components of the BBB accompany natural aging causing cognitive decline and dementia [1,9]. These disruptions become more detrimental when exposed to a second hit, such as inflammation [48]. The morphology and rigidity of erythrocytes change with age, altering their transport within the capillary bed. This additionally triggers downstream biological events, such as the release of reactive oxygen species and hemoglobin, potentially compromising the BBB [49]. Particularly susceptible to mechanical damage caused by senile erythrocytes are the capillary blood vessels in the brain regions that are responsible for adult neurogenesis. Vascular cells are known to play a prominent role in regulating the proliferation of adult neural precursors. Dense clusters of dividing cells were found to be anatomically close to the vasculature, especially in the capillaries. Contacts between the subventricular zone SVZ neuronal precursors and blood vessels are unusually permeable and frequently devoid of astrocyte and pericyte interferences, suggesting that blood-derived cues are gaining direct access to adult neural precursors and their progeny. This vasculature also provides the substrate for new neuron migration after injury in the adult striatum [47]. The proper functioning of SVZ is responsible for the synaptogenesis and reorganization of motor memory networks [50,51]. Aging-related changes in BBB permeability also cause the accumulation of blood-derived proteins and neuronal debris in the brain, which additionally damage the BBB. As a result, there is a progressive disintegration of metabolic processes in the brain, especially energy metabolism [1,9].

Increased brain or muscular activity can result in a rapid decrease in serum phosphate (reactive hypophosphatemia) due to the intensive uptake of phosphate into cells that maintain increased intracellular metabolism. Similarly, hypophosphatemia is observed in cases of increased blood glucose levels and respiratory alkalosis. It results in the intracellular depletion of phosphate, which, in turn, leads to a decrease in metabolites such as ATP and 2,3DPG.ATP is essential for the maintenance and activity of neural and muscular cells; however, the depletion of ATP leads also to the destruction of red blood cells, anemia, and reticulocytosis. Chronic hypophosphatemia was shown to cause respiratory failure and respiratory muscle weakness, and rhabdomyolysis. All these, taken together, contribute to extreme susceptibility to fatigue [52].

## 7. Aging-Related Dysfunction of Phosphate Signaling

The morphological and biochemical age-related changes and oxidative stress in red blood cells seem to have a leading role in organismal aging [28]. An important contributor to reduced systemic blood flow in the elderly is increased vascular stiffness and resistance due to the calcification of the muscle walls of large vessels [20]. The stiffening of the blood vessels characterizing hypertension is caused by several factors, including an imbalance of osteotropic hormones, endothelial dysfunction, and impaired vasodilation [20].

Systemic phosphate distribution depends solely on blood transport which is heavily affected by aging processes. Red blood cells (RBC) have a very specific cellular structure and physiology, which determines their short longevity [28,29,30,31,53]. The morphology of senescent RBCs is characterized by cell shrinkage and membrane blebbing, which makes them fully inefficient. Defective RBCs are removed from circulation by macrophages of the mononuclear phagocytic system when passing through the splenic and hepatic sinusoids. The phagocytosis of RBCs can be mediated by the phospholipid component of the cell membrane, phosphatidylserine (PS), and transmembrane protein, integrin, also called the Cluster of Differentiation 47 (CD47). Both play a key role in cell cycle signaling, specifically in apoptosis. CD47 acts as an antiphagocytic signal and is confined to the external surface of the cell membrane in matured erythrocytes. However, the surface protective layer is progressively lost, releasing prophagocytic signals of PS. The net balance between PS and CD47 in an RBC determines whether or not it is destroyed by the macrophages [25].

The only store of inorganic phosphate is bone tissue, whose osteocytes control and balance the quantity of phosphorus and calcium ions in circulation [2,3]. Osteocytes are the most numerous cells in mature bone that have the potential to live as long as the organism itself [54]. Osteocytes have a role in mechanotransduction and are assumed to modulate activity associated with remodeling and bone turnover [54]. Bones are mechanically stimulated during every motor activity. The sensitivity of bone mechanosensitivity declines with age, leading to a growing deficit of phosphate-calcium homeostasis, culminating in hypoactivity and osteoporosis [54]. Osteocytes are bone cells found in the most abundant mature bone [18]. Their lifespan is comparable to that of an organism, and they are not capable of proliferating. They coordinate the levels of phosphate and calcium ions in circulation, ensuring systemic homeostasis and energy balance. The dysfunction or deficiency of the phosphate-calcium tandem causes disturbances in the functioning of both the nervous and muscular systems [2,3,11]. Importantly, in physiological conditions, the phosphate-calcium synergy is tuned by energy metabolites, including glucose and oxygen, as well as by physical activity, which stimulates the phosphate release from bones [18].

In the aging body, the transport and distribution of phosphate decreases rapidly, possibly as a result of the impaired proliferation of hematopoietic stem cells. The age-related deficiency of the histone deacetylase SIRT6 impairs hematopoietic stem cell proliferation and the ability for self-renewal [55]. SIRT6 can bind NAD, allowing it to act as an NAD sensor [56]. In senescent bone marrow, NAD deficiency is probably the main factor limiting SIRT6 activity. Rising deficiency in new erythrocytes finally disturbs and terminates organismal phosphate metabolism and function. NAD deficiency impacts cellular homeostasis and glucose and lipid metabolism, thus affecting diseases such as diabetes, obesity, heart disease, and cancer [57]. So far, research on treatments targeting Sirt6 deficiency has been very promising. It seems, however, that we should also focus on aging-related NAD deficiency [1].

The ability of ATP to stimulate nitric oxide production in endothelial cells makes RBC aging the main determinant of blood flow [58]. The ATP release from RBCs and their vitality are closely dependent on their cellular antioxidant defense status. Erythrocytes are particularly vulnerable to reactive oxygen species [59]. The RBC’s ability to recover from oxidant insults depends upon glucose-6-phosphate dehydrogenase (G6PD) activity. G6PD is an enzyme that is found in the cytoplasm of all cells in the body and playsa vital role in the prevention of cellular damage from reactive oxygen species [59]. Enucleated and mitochondria-depleted erythrocytes are particularly susceptible to oxidative stress. It is well documented that G6PD and NADPH-deficient senescent RBCs are subjected to high oxidative stress and, hence, the stiffening of the cell membrane due to the oxidation of important membrane proteins, which escalate their aging [58]. Studies on heterochronic blood exchange and plasma manipulation have established a causal connection between blood morphological changes and brain health and function. Their impact depends on erythrocyte viability, deformability, and biochemistry [49]. Importantly, brain health and function can be recovered through the dilution of old plasma with saline plus albumin [49].

Hyperphosphatemia is usually a consequence of a decreased level of ionized calcium, which leads to a decrease in the level of PTH, and, consequently, an increase in intestinal calcium absorption with a simultaneous decrease in vitamin D synthesis. This leads to hypercalcemia, which is accompanied by digestive and hormonal disorders and can lead to changes in bone metabolism (osteoporosis) and nervous and mental symptoms. An increase in the level of calcium above the physiological threshold causes a change in the excitability threshold of neurons and muscle fibers. Pathophysiological levels of calcium block sodium channels, causing smooth and skeletal muscle hypotonicity, resulting in weakness and low tone of skeletal muscles. Consequently, the excitability of the nerves and muscles decreases. Neurological disorders due to elevated calcium levels include drowsiness, disorientation, hallucinations, stupor, and even coma. Several factors can affect high calcium levels, such as hypertrophy of the thyroid gland, adrenal insufficiency, but also prolonged immobilization. 

This process of aging can be initiated by changes in hematopoiesis. Blood cells are produced from a limited number of hematopoietic stem cells (HSCs), which reside in the bone marrow. The stability of the bone marrow microenvironment is essential for the maintenance of fully functional hematopoiesis throughout life [60]. The bone marrow microenvironment exerts its regulatory activity on HSCs and their progeny. The deregulation of the bone marrow architecture occurs in both acute and chronic infections as well as aging. Acute or chronic stress deregulates hematopoiesis [60]. The efficiency of erythropoiesis is under the control of nervous, hormonal, and many local and systemic trophic factors. The effectiveness and efficiency of all of the above controls decrease with age. Erythrocytes in the elderly have noticeable morphological and functional changes. Their lifespan decreases, falling even to 10–11 days. Thus, the efficiency of the erythropoietic process becomes so low that it cannot meet the needs of phosphate transport. As a consequence, the elderly may experience a breakdown in life processes as a result of hypophosphatemia. The quality and viability of mature erythrocytes might be significantly affected by the decreasing intracellular level of NAD in reticulocytes with the number of divisions. In the last phase of maturation, erythrocytes still have a nucleus and mitochondria. However, the proper functioning of these cellular organelles is limited by the intracellular level of NAD [61], which rapidly decreases with age [1]. NAD deficiency causes the inhibition of ATP production and a decrease in NAD-dependent repair processes, which intensifies hematopoietic insufficiency and, consequently, accelerates the aging of the body. Research on these phenomena began very recently, and at the moment, it is difficult to assess to what extent we are able to slow down or control the aging process [62].

The most intriguing but poorly understood pathomechanism of aging is the growing disturbance in the interaction between the internal and external environment. This interaction takes place in the bones, which integrate physical activity with organismal energy metabolism. Toward this end, the bone marrow niches regulate hematopoiesis and osteoblastogenesis. Factors influencing this process occur through cellular, physical, and chemical interactions within the bone marrow microenvironment [63]. Recently it has been documented that an inverse relationship between bone mineral density and marrow adipocytes and hematopoiesis exists [63]. Noteworthy is the fact that bone mineral density is controlled by both the mechanical load and hormonal signaling. The activity of aged individuals is characterized by hypoactivity, which strikes both muscular glucose storage as well as bone marrow adipocyte tissue (MAT). Consequently, skeletal homeostasis is actively mediated through MAT’s interaction with osteoblasts [63]. Endosteal adipocytes are rare in neonates; these cells steadily accumulate throughout their lifespan and occupy a greater proportion of the bone marrow cavity in the axial skeleton with aging. Particularly, hypoactivity is associated with increased MAT and low bone mass [63]. Recent studies have demonstrated increases in bone mineral density along with significant decreases in femoral MAT with physical activity. During aging, the bone marrow cavity gradually becomes filled with adipocytes while the bone is lost. Concomitantly, levels of the growth hormone also decline. In humans with growth hormone deficiency, adipocytes rapidly accumulate within the bone marrow cavity [63]. The decline in estradiol and dihydrotestosterone levels, as seen with aging, also increases the differentiation of MSCs into adipocytes impairing hemopoiesis. Simultaneously, elevated levels of circulating glucose become neurotoxic and damage the neural network that is responsible for metabolic homeostasis, which together opens the vicious circle of aging.

In summary, bone tissue plays a vital role in the body’s environmental adaptation. The bone system forms an interface between the organism and the environment. Bone metabolism combines physical activity with adaptive changes in the internal environment of the body, which is necessary for its survival. The operation of the adjustment mechanisms is based on the control of the phosphate-calcium metabolism. Phosphate-calcium signaling participates in the primary mechanism of controlling homeostasis and its recovery after exercise-induced disturbances. Phosphates play an important role in the regulation of energy metabolism both by regulating postprandial glucose storage in muscles, liver, and adipose tissue, as well as the distribution and supply of energy fuel and metabolites to the brain and skeletal muscles. Age-related disorders of phosphate and calcium metabolism result in the escalation of aging processes and metabolic diseases of old age. Understanding these mechanisms is essential for introducing effective methods for treating these diseases and controlling the aging process itself.
